# Green synthesis of nanohydroxyapatite with *Elaeagnus angustifolia L*. extract as a metronidazole nanocarrier for in vitro pulpitis model treatment

**DOI:** 10.1038/s41598-024-65582-4

**Published:** 2024-06-26

**Authors:** Sepideh Sarfi, Ehsaneh Azaryan, Mohammad Yahya Hanafi-Bojd, Fariba Emadian Razavi, Mohsen Naseri

**Affiliations:** 1 Student Research Committee, Birjand University of Medical Sciences, Birjand, Iran; 2 Department of Immunology, School of Medicine, Birjand University of Medical Sciences, Birjand, Iran; 3 Cellular and Molecular Research Center, Birjand University of Medical Sciences, Birjand, Iran; 4 Department of Pharmaceutics and Pharmaceutical Nanotechnology, School of Pharmacy, Birjand University of Medical Sciences, Birjand, Iran; 5 Dental Research Center, Faculty of Dentistry, Birjand University of Medical Sciences, Birjand, Iran; 6 Cellular and Molecular Research Center, Department of Molecular Medicine, Birjand University of Medical Sciences, Birjand, Iran

**Keywords:** Drug delivery platforms, Green synthesized, Nanohydroxyapatite, Dental pulp stem cells, Anti-inflammation, Pulpitis, Nanobiotechnology, Immunology, Molecular biology, Stem cells

## Abstract

The aim of this study is to introduce a dental capping agent for the treatment of pulp inflammation (pulpitis). Nanohydroxyapatite with *Elaeagnus angustifolia L.* extract (nHAEA) loaded with metronidazole (nHAEA@MTZ) was synthesized and evaluated using a lipopolysaccharide (LPS) in vitro model of pulpitis. nHAEA was synthesized through sol–gel method and analyzed using Scanning Electron Microscopy, Transmission Electron Microscopy, and Brunauer Emmett Teller. Inflammation in human dental pulp stem cells (HDPSCs) induced by LPS. A scratch test assessed cell migration, RT PCR measured cytokines levels, and Alizarin red staining quantified odontogenesis. The nHAEA nanorods were 17–23 nm wide and 93–146 nm length, with an average pore diameter of 27/312 nm, and a surface area of 210.89 m^2^/g. MTZ loading content with controlled release, suggesting suitability for therapeutic applications. nHAEA@MTZ did not affect the odontogenic abilities of HDPSCs more than nHAEA. However, it was observed that nHAEA@MTZ demonstrated a more pronounced anti-inflammatory effect. HDPSCs treated with nanoparticles exhibited improved migration compared to other groups. These findings demonstrated that nHAEA@MTZ could be an effective material for pulp capping and may be more effective than nHAEA in reducing inflammation and activating HDPSCs to enhance pulp repair after pulp damage.

## Introduction

Pulpitis, characterized by inflammation of the dental pulp, is increasingly prevalent due to improper brushing and unhealthy dietary habits^1^. Oral bacteria that contribute to the formation of cavities play a significant role in initiating pulpitis^2,3^. Untreated pulpitis may cause tooth necrosis^4^ and because pulp is vital for tooth function, it is essential to treat it^5^. Pulpitis treatment often involves replacing the pulp with substances like cement, which can cause teeth to lose pulpal sensibility to hot/cold stimulation and secondary infections. Therefore, using restorative therapeutic methods for pulpitis is crucial. Vital pulp therapy (VPT) aims to maintain the vitality and function of the dentine–pulp complex by stimulating reparative dentin formation to maintain the tooth as a functional unit. The focus is directed toward the preservation of the permanent tooth, based on the assumption that tooth pulp has an innate capacity for repair in the absence of microbial contamination. Direct and indirect pulp capping, which are less invasive methods of VPT, strives to maintain the integrity of the pulpal tissue. These techniques aim to promote pulp healing using biocompatible materials for the differentiation of human dental pulp stem cells (HDPSCs) to odontoblasts and then reparative dentin^6^.

Human dental pulp stem cells (HDPSCs) are a population of highly proliferating mesenchymal stem cells. The ability of these cells to differentiate into various kinds of cell lines, such as odontoblast that produce dentin, and the simplicity that their source can be accessed, allow them to effectively contribute to the advancement of tissue engineering and regeneration of a variety of tissues, such as dental pulp and dentin complex^7–11^. Based on the studies, the best strategy to treat pulpitis could be to control the inflammation of HDPSCs, guide them to migrate inwards, differentiate them into dentine cells, and increase their mineralization capacity. Based on this strategy, the use of pulp-capping materials that can stimulate the secretion of various signaling molecules to stimulate the mineralization of HDPSCs and regulate inflammation is recommended. One of the popular materials for pulp-capping is hydroxyapatite (Hap)^12^.

Calcium and phosphate are primary components in teeth. Calcium and phosphate-based ion delivery systems, like HAp (Ca_10_(PO4)_6_(OH)_2_), by augmenting these ions in the oral milieu, exhibit therapeutic potential like fostering osseous growth proximal to osteogenic cells, thereby fostering bone differentiation^13^. nHAp particles will disintegrate in the stomach so they are safe to be eaten. One of the methods for synthesizing nHAp is the synthesis through biological and green materials^14–16^. Green synthesis is a method that uses natural processes and provides an environmentally friendly alternative. This technique creates nanoparticles (NPs) clean and safely using low-energy, naturally derived ingredients. Bacteria, fungi, algae, and specific plants are environmentally friendly substrates for green synthesis^17^. Synthesis using intermediate biological materials can be done cost-effectively, in large quantities, and without significant toxic effects^18^. Accordingly, we synthesized nHAp utilizing *Elaeagnus* *angustifolia* *L*. extract (nHAEA). Herbal extracts like EA are utilized in research due to their impact on bone formation and inflammation. EA is from the *Elaeagracea* family^19,20^. Tannins and flavonoids in EA have anti-inflammatory characteristics and boost angiogenesis activities^21–23^. EA contains phytoestrogens, which are natural estrogens. Estrogens can encourage bone formation, so EA has bone-strengthening effects. Also, Phenolic compounds of EA can enhance alkaline phosphatase activity, stimulating osteoblast differentiation and mineralization^19^.

Nanohydroxyapatite is renowned for its high surface-to-volume ratio, making it an ideal candidate for the loading of various antibiotics^24^ and because of its positively charged surface's interaction with deprotonated carboxyl groups alongside the negatively charged groups found in antibiotic compounds, nHAp has a high adsorption capacity for these kinds of drugs^13^. So, HAp is an appropriate carrier for the controlled release and a vehicle for the local administration of drugs^25–28^.

Metronidazole (MTZ), an antibiotic belonging to the nitro-5-imidazole family^29^, can inactivate germs in the oral cavity, and also be used as an anti-inflammatory medicine^1^. MTZ inhibits the production of reactive oxygen species (ROS) and reduces inflammatory cytokines like IL-6 in human periodontal ligament cells (hPDLCs), triggered with lipopolysaccharide (LPS)^30^. Due to its antibacterial properties and ability to reduce inflammatory cytokine levels, MTZ is potentially an efficient pulpitis treatment. However, MTZ utilization is restricted and difficult due to its short half-life.

NPs containing antibiotics have been created for local treatment because controlling the antibiotic dose and its diffusion rate leads to a higher concentration of the drug in the target site with a lower dose, increasing the performance of HDPSCs and promoting regeneration in the environment while minimizing the toxic side effects of systemic antibiotic use^31^.

The tooth’s defense and healing processes are connected. The host's immune responses react to bacteria, leading to inflammation^30^. Inflammation and repair are closely related^3^. Persistent and severe inflammation harms the pulp, mild to moderate inflammation promotes pulp regeneration. The pulp capping materials utilized must adjust the equilibrium between inflammation and regeneration and encourage HDPSC differentiation. Employing a technique capable of managing inflammation without impeding regeneration can serve as a viable substitute for conventional materials^12^. The study will evaluate the control of inflammation, differentiation, and mineralization of cells to develop nanoparticles that can effectively manage inflammation, repair and rebuild the pulp-dentin complex, and offer a suitable alternative for common pulp therapy materials.

**Figure 1:**
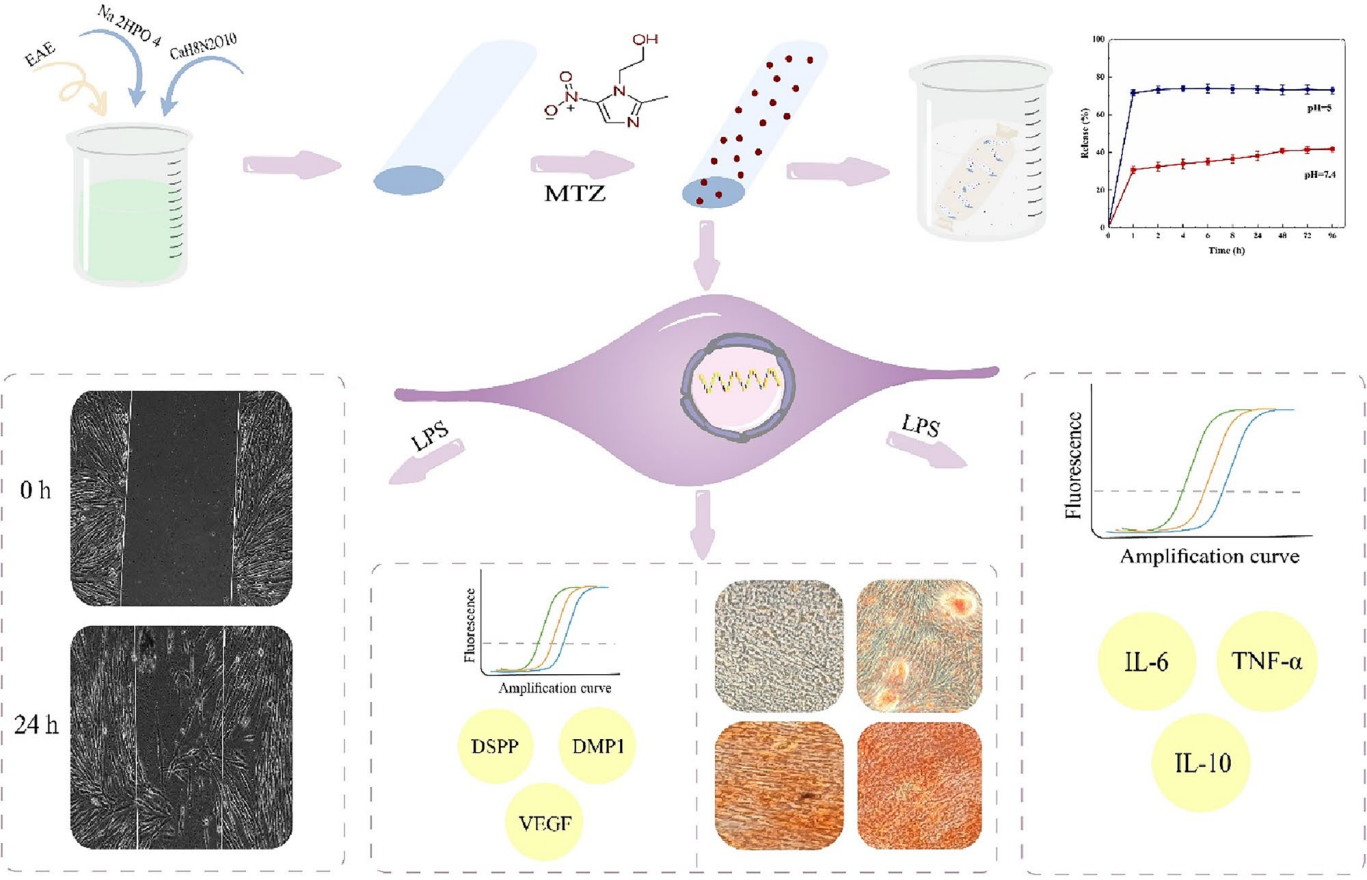
Schematic illustration of the study.

Therefore, in this project, nanohydroxyapatite synthesized using *Elaeagnus angustifolia* *L*. extract (nHAEA) was utilized for the controlled delivery of metronidazole to dental pulp stem cells. Additionally, we designed a model of LPS-induced inflammation in HDPSCs and examined the impact of nHAEA@MTZ on the inflammatory response and migration, also osteogenic differentiation of HDPSCs was assessed with nHAEA@MTZ (Fig. 1).

## Material and methods

### nHAEA synthesis

All experimental protocols were approved by the ethical committee of Birjand University of Medical Science (ethical number IR.BUMS.REC.1402.013). The nHAEA was synthesized in accordance with our earlier research^32^. *Elaeagnus angustifolia L*. pulps were pulverized after being dried. The maceration method was then used to mix about 40 g of powder, with 320 mL of methanol and 80 mL of distilled water as the solvent. To produce a solid crude extract, the solution was filtered and concentrated in a rotating vacuum. The extract was kept at 4 °C. Calcium nitrate tetrahydrate and diammonium hydrogen phosphate (both from Sigma-Aldrich, USA) were dissolved in deionized water. The calcium nitrate tetrahydrate solution was added to the extract solution (solved with distilled water) and agitated for 30 min at 50 °C. The diammonium hydrogen phosphate solution was then added drop by drop (1 mL/min) to the prepared solution. The pH was then increased to 11 by adding NaOH. The mixture was stirred for 90 min at 50 °C. Following centrifugation, the resulting mixture was washed three times with ethanol and distilled water.

### Characterization of nHAEA

We used Fourier transform infrared (FTIR) to assess functional groups. Morphological examinations and characterization of the dimensions and configuration of nHAEA were conducted using a Scanning Electron Microscope (SEM, BRNO-Mira3 model, Tescan Co., Czechia) and a Transmission Electron Microscope (TEM, EM10C-100 kV device, ZEISS Co., Germany). Additionally, a comprehensive analysis of the surface area and porosity of the NPs was performed employing the Brunauer–Emmett–Teller (BET) isotherm approach, utilizing the BElSORP Mini II BET apparatus (Microtrac Bel Corp Co., Japan). Before analysis, the NP samples underwent a two-hour degassing process at 120 °C to eliminate potential contaminants and moisture from the NP surfaces.

### Drug loading onto the nanoparticle

To determine the lambda max (λ_max_) of MTZ (Sigma-Aldrich, USA), a stock solution of 5 mg MTZ in 1 mL of distilled water was produced. Serial dilutions were performed, and absorbance spectra were scanned using a Biotek Epoch spectrophotometer (Winooski, VT, USA), resulting in λ_max_ at 319 nm. These results were used to create an MTZ calibration curve. Two ratios were used for drug loading into NP: a 1:1 ratio with 10 mg of MTZ and 10 mg of NP in 1 mL of distilled water, and a 2:1 ratio with 20 mg of MTZ and 10 mg of NP in the same amount. The mixtures were shaken for 24 h before being centrifuged at 12,000 rpm for 20 min to separate unloaded MTZ from MTZ-loaded nanohydroxyapatite (nHAEA@MTZ sediment). The supernatant was serially diluted and measured at λ_max_. The MTZ content was calculated using the MTZ solution calibration curve. The drug loading content (DLC) and drug loading efficiency (DLE) were then calculated using equations (Eqs. 1 and 2, respectively).

1 $${\text{DLC}} = \frac{{\text{weight of loaded MTZ in NP }}}{{\text{weight of MTZ and NP}}} \times 100$$

2 $${\text{DLE}} = \frac{{\text{weight of loaded MTZ in NP }}}{{\text{weight of feeding MTZ}}} \times 100$$

Sediment was dissolved in the desired solvent for subsequent tests**.**

### Membrane diffusion drug release study

Over a four-day period, NP in vitro drug release was investigated using the dialysis bag technique at two different pH values. A dialysis bag with a molecular weight cut-off (MWCO) 12,400 Daltons (Sigma-Aldrich, USA), was employed. The membrane was submerged in each buffer for approximately 30 min to remove any remaining preservatives. A magnetic stir bar was immersed in 200 mL of phosphate-buffered saline (PBS) (pH = 7.4) and sodium acetate buffer (SAB) (pH = 4.5) in a compartment, and 1 mL of nHAEA@MTZ was added to the membrane and put into receptor compartments. At 1, 2, 4, 6, 8, 24, 48, 72, and 96 h, 1 mL samples were taken from the buffers, and fresh buffer was added immediately after sampling the previous buffer. To calculate drug release %, each buffer's λ_max_ was measured using a spectrophotometer, and a calibration curve was created. Data were subsequently analyzed using GraphPad Prism 9 (GraphPad Software, USA).

### HDPSCs isolation and characterization

Patients (20–25 years old) in the Dental Center of Imam Reza Hospital in Birjand (Iran) provided informed consent, and the ethical committee of Birjand University of Medical Science approved the collection of human adult's third molars (ethical number: IR.BUMS. REC.1399.090). Before participation, everyone gave written consent. All protocols were conducted following the relevant regulations and guidelines^33^. After transferring the dental sample to the Laboratory of Research at Birjand University of Medical Sciences, the process of extracting stem cells from the pulp was carried out within a maximum of 24 h using the enzymatic digestion method (collagenase 1 enzyme). To achieve this, after the tooth was fractured under sterile conditions, the pulp tissue was extracted and cut into small pieces using a surgical blade No. 10. The pulp pieces were then suspended in falcon tubes containing phosphate buffer and 100 U/mL collagenase type I enzyme (BIO-IDEA, Iran), and incubated at 37 °C for 1 h. Following enzymatic digestion, the enzyme was inactivated and neutralized by adding a sufficient amount of complete culture medium with 20% fetal bovine serum (FBS). The tubes were centrifuged at 2000 rpm and 30 °C for 5 min. The floating materials were removed from the tube surfaces, and the pulp tissue and settled cells were transferred to T25 flasks containing Dulbecco's modified Eagle’s medium/F12 (DMEM/F12; Betagen, Iran) enriched with 10% FBS, 100 U/mL penicillin, 100 µg/mL streptomycin, and 0.25 µg/mL amphotericin B. The flasks were then incubated at 37 °C and 5% CO_2_.

In this protocol, after approximately 3–5 days, spindle-shaped cells can be observed around the tissues. These cells are the stem cells that, following the need for more nutrients in the FBS-enriched culture medium, migrate out of the pulp tissue according to the cell scape phenomenon. The culture medium was changed every 3 days to allow 70% of the flask bottom to be covered with cells. When the cell confluency reached 70%, the cells were passaged and characterized^34^. HDPSCs were grown in DMEM (Grand Island, USA) with 1% penicillin–streptomycin (Sigma-Aldrich, USA) and containing 10% FBS (Grand Island, USA) in a cell culture incubator at 37 °C with 5% CO_2_. The HDPSCs from passages 3–6 were used in the following studies. Cells were detachment using a trypsin–EDTA (Grand Island, USA) solution and grown in fresh flasks or plates.

### Cell inflammation by LPS

A 5 mg/mL stock solution of LPS was made by dissolving 1 mL of LPS powder purchased from Sigma-Aldrich, USA in distilled water. Then, a 500 µg/mL stock solution was produced in an FBS-free medium. 5 × 10^5^ cells were plated in a 60 mm petri dish, incubated overnight, and treated with either nHAEA or nHAEA@MTZ. Both NPs were produced in an FBS-free medium at a concentration of 50 mg/mL. After a 1 h exposure to the NPs, cells were stimulated with 1 µg/mL LPS for 24 hours^35^. The resultant LPS-induced HDPSCs were called inflammatory human dental pulp stem cells (iHDPSCs).

### Cell viability assay

The 3-(4-dimethylthiazol-2-yl)-2,5-diphenyl tetrazolium bromide (MTT) colorimetric test was used to determine the optimum non-toxic dose of nHAEA and nHAEA@MTZ on HDPSCs. A 96-well plate of HDPSCs was seaded with 10^4^ cells per well. After an overnight incubation period, HDPSCs received treatments with LPS and then NPs concentrations of 5, 10, 25, and 50 µg/mL. 1, 3, and 7 days following treatment, 10% of the culture medium, MTT (Sigma-Aldrich, USA) in PBS, was added to each well, and the plate was incubated for 4 h at 37 °C in the dark. Because of the lipophilic side groups and positive charge, MTT can enter living eukaryotic cells, where it is converted to water-insoluble formazan. The quantity of formazan dye generated and the number of metabolically active cells are directly connected. After removing the supernatant, 100 µL of Dimethylsulfoxide (DMSO) (Merck, Germany) was added to the formazan, producing a blue-purple color. A scanning spectrophotometer was used to determine the optical absorbance of the samples at 570 and 630 nm.

### HDPSCs migration

A scratch test was used to evaluate the HDPSCs' capacity for migration. HDPSCs were seaded on 12-well plates (SPL, Korea) involving 2 × 10^5^ cells per well. A 200 µL tip was used to create a wound in each plate. Cell migration was observed via inverted light microscope (Olympus, Japan) 0 and 24 h after treatment with 50 µg/mL nHAEA@MTZ with or without 1 µg/mL LPS and 50 µg/mL nHAEA with 1 µg/mL LPS. ImageJ was used to determine the wound healing area, and GraphPad Prism was used to analyze information.

### Odontogenic differentiation of HDPSCs

HDPSCs were seeded at a density of 2 × 10^5^ cells per well in a 6-well plate, utilizing the previously mentioned standard cell culture medium rather than the odontogenic medium. Upon attaining an approximate 70% confluence, the wells were treated with either nHAEA or nHAEA@MTZ, except for the control group. Subsequently, the cells were cultured for 21 days, with regular refreshment of the culture medium every 3 days throughout the incubation.

### Alizarin red S staining

HDPSCs were plated into 24-well plates (SPL, Korea) in normal mediums. HDPSCs were treated with NPs when reaching 70% confluence, while another group remained untreated as the control. The positive control group consisted of cells exposed to an osteogenic induction medium, including 10 mM β-glycerophosphate, 10 nM dexamethasone, 50 µM L-ascorbic acid (all obtained from Sigma-Aldrich, USA), 10% FBS, and 1% penicillin/streptomycin, supplemented in DMEM. All cells underwent a 14-day culture period. Every 3 days during the incubation period, the culture medium was replaced with a fresh medium. On day 14, the cells were washed twice with PBS, fixed for 1 h at room temperature with ice-cold 70% ethanol, washed in distilled water, then stained for 30 min at room temperature with Alizarin red S (ARS) dye (40 mM, pH 4.2) (Sigma-Aldrich, USA). After 5 rounds of washing the cells with deionized water to get rid of the excess dye, 1 mL of deionized water was added to each well, and qualitative analysis with an inverted light microscope was conducted. After that, incubating cells with 10% cetylpyridinium chloride for 15 min accompanied by agitation for calcium ion desorption, and quantitative analysis was done by measuring the absorbance of the solution at 570 nm.

### Quantitative real-time polymerase chain reaction (qPCR) analysis

After 21 days of odontogenic differentiation and 24 h of inflammatory treatment, HDPSCs were washed twice in PBS before total RNA extraction with TRIzol Reagent (Invitrogen, Thermo Fisher Scientific, USA) according to the manufacturer's instructions. The samples' quality was verified with a NanoDrop spectrophotometer (Biotek Epoch, USA) at 260/280 and 260/230 nm wavelengths. The RNA was then transcribed into cDNA using the Pars Tous cDNA Production Kit. qPCR was used to examine the expression levels of TNF-α, IL-6, IL-10, VEGF-A, DSPP, and DMP1 mRNA. The study used the 2 ^− ΔΔCT^ approach to normalize target gene expression against the endogenous control, GAPDH. SYBR Green PCR Master Mix (Amplicon, Denmark) was used for the qPCR analysis. The Applied Biosystems ABI Step One Plus real-time PCR device was used for amplifying cDNA. Table 1 displays the primer sequences that were used in this study.

**Table 1:** Sequences of primers used for real-time PCR.

Gene	Forward primer	Reverse primer	Product (bp)
IL-6	AGACTTGCC TGGTGAAAA TCA	GCTCTGGCTTGT TCCTCACT	100
TNF-α	CACAGTGAAGTGCTGGCAAC	ACATTGGGTCCCCCAGGATA	357
IL-10	GACATCAGGGTGGCGACTC	CAATAAGGTTTCTCAAGGGGCTG	107
DMP1	CAGTGCCCAAGATACCACC	TGCATTCCCTCATCGTCCA	180
DSPP	TTCCGATGG GAGTCCTAG TG	TCTTCTTTCCCA TGGTCCTG	144
VEGF-A	AGGGCAGAA TCATCACGA AGT	AGGGTCTCGATT GGATGGCA	75
GAPDH	CGAACCTCT CTGCTCCTC CTGTTCG	CATGGTGTCTGA GCGATGTGG	83

### Statistical analysis

The statistical analysis was done using GraphPad Prism applications, Version 9, and all experiments were done in triplicate. We calculated the mean and standard deviation (SD). A one-way analysis of variance (ANOVA) with *p* < 0.005 was used to determine the statistical significance of the differences between the research groups that were observed to be significant.

### Ethics approval and consent to participate

All experimental protocols were approved by a named institutional and/or licensing committee. 1. All experimental protocols were approved by the ethical committee of Birjand University of Medical Science (ethical number IR.BUMS.REC.1402.013). 2. A statement to confirm that all experimental protocols were approved by a named institutional and/or licensing committee. The Human Dental Pulp Stem Cell (HDPSC) isolation procedure was carried out in compliance with the Declaration of Helsinki and was approved by the Research Ethics Committee at Birjand University of Medical Sciences (Ethical number: IR.BUMS.REC.1399.090). The teeth were removed from healthy donors as part of their dental treatment plan. All protocols were conducted following the relevant regulations and guidelines. Before participation, everyone gave written consent. 3. Experimental research and field studies on plants including the collection of plant material complying with relevant guidelines and regulations for plant ethics.

## Result

### Characterization of nHAEA and physical properties

**Figure 2:**
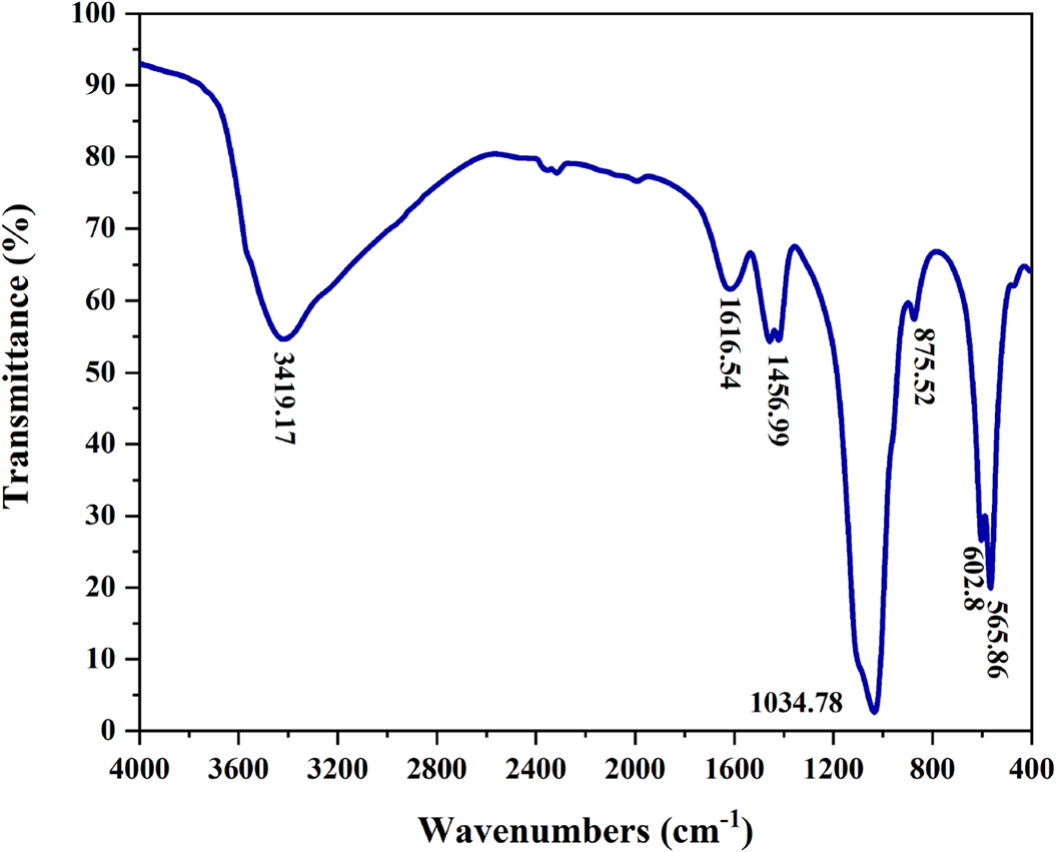
FTIR spectra of nHAEA.

FTIR: The functional groups and molecular linkages of nHAEA nanoparticles were identified through FTIR analysis within the range of 400–4000 cm^−1^, as depicted in Fig. 2. Based on the spectrum analysis, it has been determined that the nHAEA includes all of the necessary functional groups and is free of impurities. Consequently, it can be inferred that the HAp nanoparticles have been successfully synthesized in conjunction with the extract of *Elaeagnus angustifolia L*.

**Figure 3:**
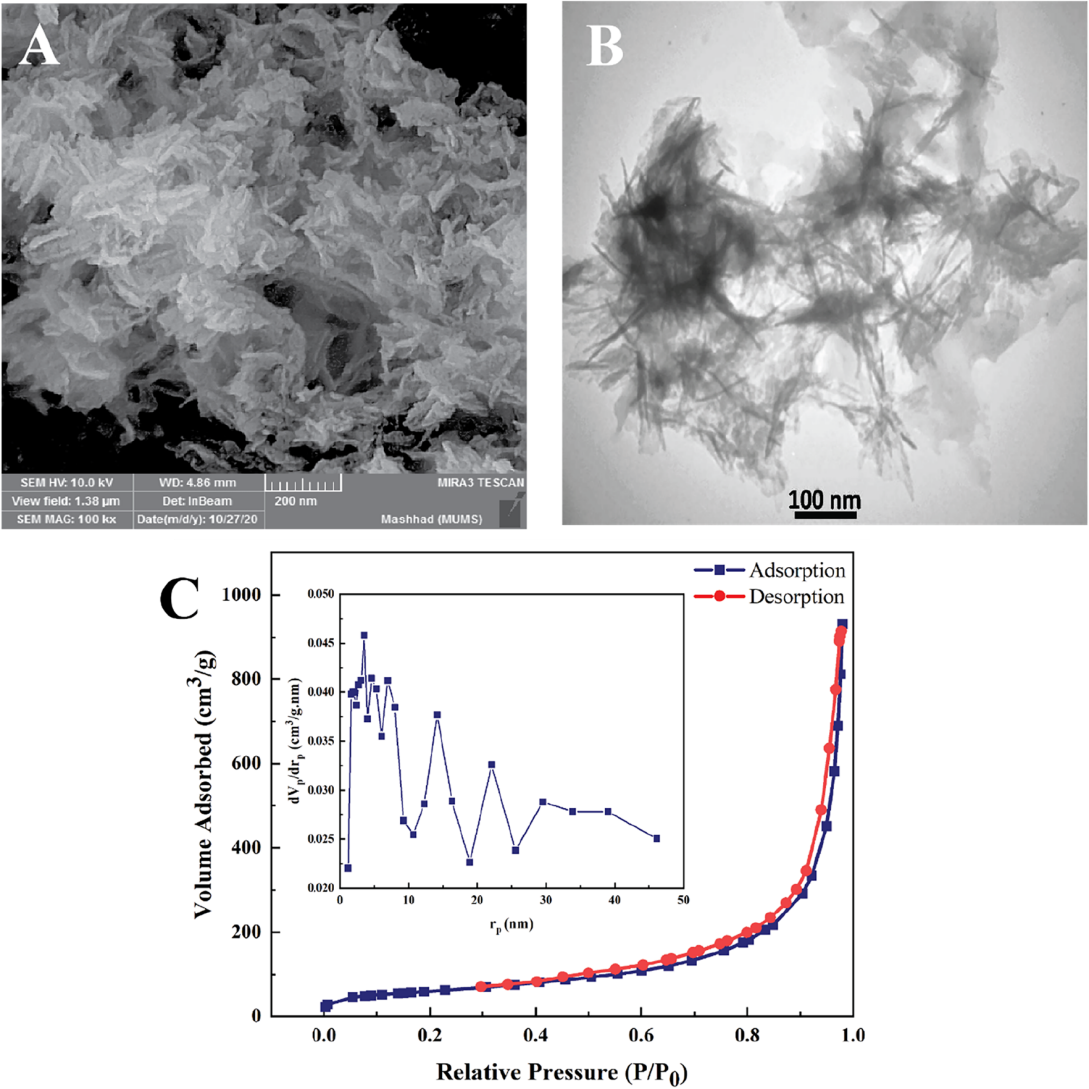
(**A**) SEM analysis; (**B**) TEM analysis; (**C**) BET analysis, and BJH plot of the nHAEA.

Morphological analysis: The morphological characteristics of nHAEA nanoparticles were studied using SEM and TEM. The results of the analysis are shown in Fig. 3A and B, demonstrating that the nHAEA particles have a nanorod-like structure with a width of approximately 17–23 nm and a length of 93–146 nm.

BET analysis: Describing the characteristics of porous solids is necessary for structural elements in delivery system design. Total pore volume (V_m_), specific surface area (S_BET_), and Average pore diameter (D_p_) of NPs are checked using the nitrogen gas adsorption–desorption method and BJH analysis. Figure 3C depicts the BET analysis, which was based on the nitrogen gas adsorption–desorption rate measurement on the surface of nHAEA. According to the IUPAC classification, the nitrogen adsorption–desorption exhibited type IV isotherms, which are common for mesoporous structures and have a hysteresis loop. Results are shown in Table 2.

**Table 2:** Surface parameters of nHAEA nanoparticles.

	Surface area (m^2^/g)	Total pore volume (cm^3^/g)	Average pore diameter (nm)
nHAEA	210.89	1.44	27/312

### MTZ loading onto the nHAEA

**Figure 4:**
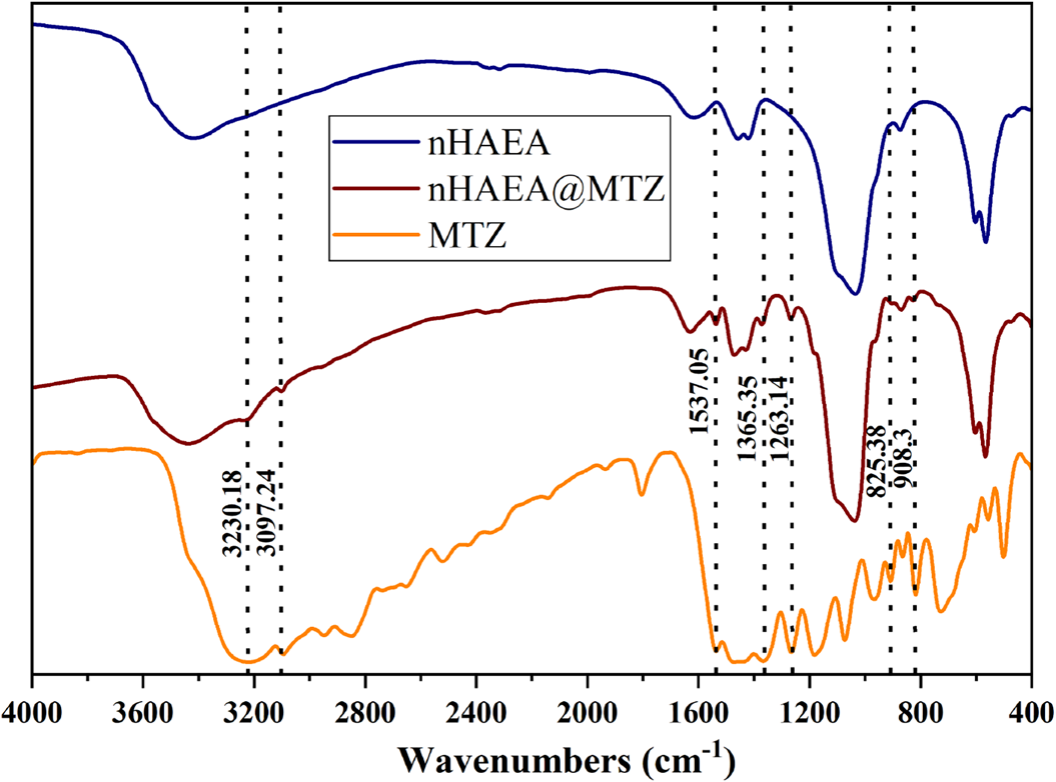
FTIR spectra of nHAEA, nHAEA@MTZ and MTZ.

Two distinct synthesis ratios were employed to ascertain the optimal mixing ratio for enhancing the loading capacity of MTZ into nanoparticles for drug delivery systems. Notably, in the context of a 2:1 ratio of MTZ to the nanoparticle, the DLC and DLE increased compared to 1:1 ratio, (Table 3). The formulated nHAEA@MTZ had a loading efficiency of 53%, which means that 53% of the added MTZ existed within the nanoparticle framework. As a result, the 2:1 ratio, which provides more content and efficacy, was selected for further investigation. To confirm the loading of the metronidazole onto nanoparticles, graphs obtained from FTIR analysis for nHAEA, nHAEA@MTZ, and MTZ were examined and presented in Fig. 4.

**Table 3:** MTZ loading content and loading efficiency of different ratios.

Drug: nanoparticle ratio	Drug loading content (w/w %)	Drug loading efficiency (%)
1:1	25.04	50.09
2:1	35.41	53.12

### Drug release

**Figure 5:**
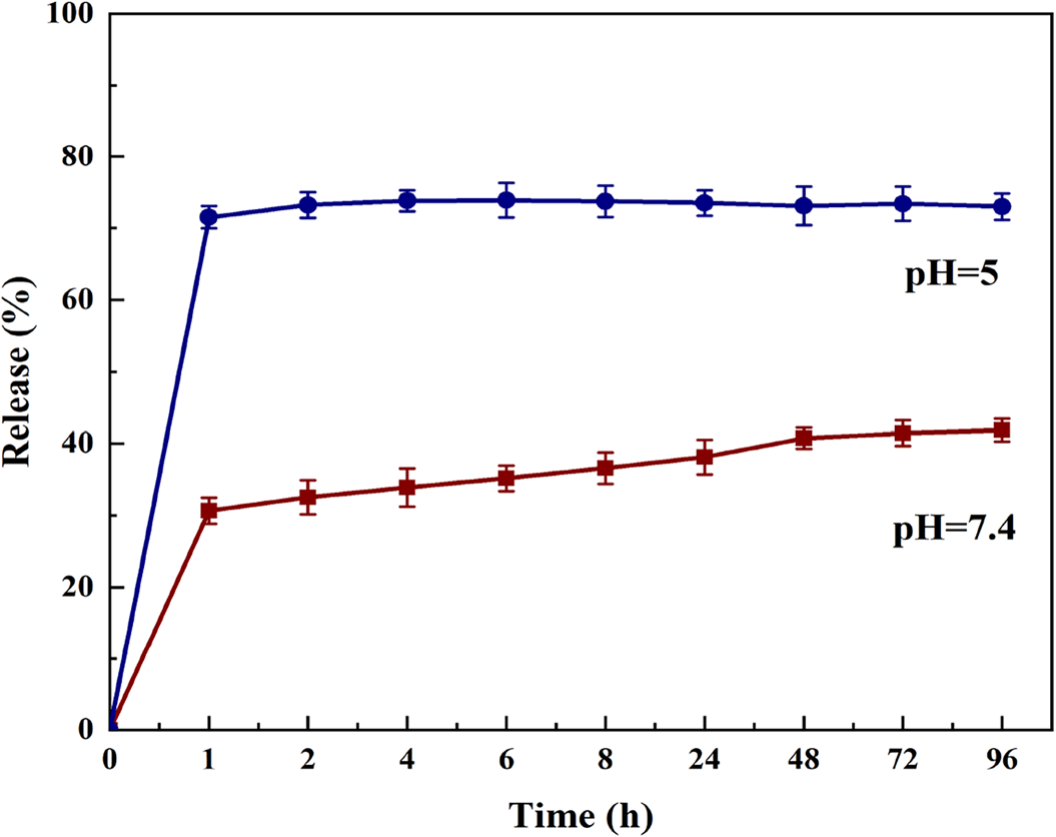
The release profile of MTZ from nHAEA in different pH buffers (n = 3).

Figure 5 shows the MTZ release curve from nHAEA at various pH levels. pH 7.4 indicates the physiological medium in its natural form, whereas pH 5 depicts day-to-day activities that involve eating sugary foods and ignoring oral care. According to the in vitro analysis, the release profile for acidic pH demonstrated an early burst release in the first hours, which was sustained at these high concentrations for the duration of the observation period, as well as a sharp release followed by an invariant release for normal pH. The nHAEA release profile at various pH levels shows how the local pH influences the overall "rate" of drug release.

### Cytocompatibility

**Figure 6:**
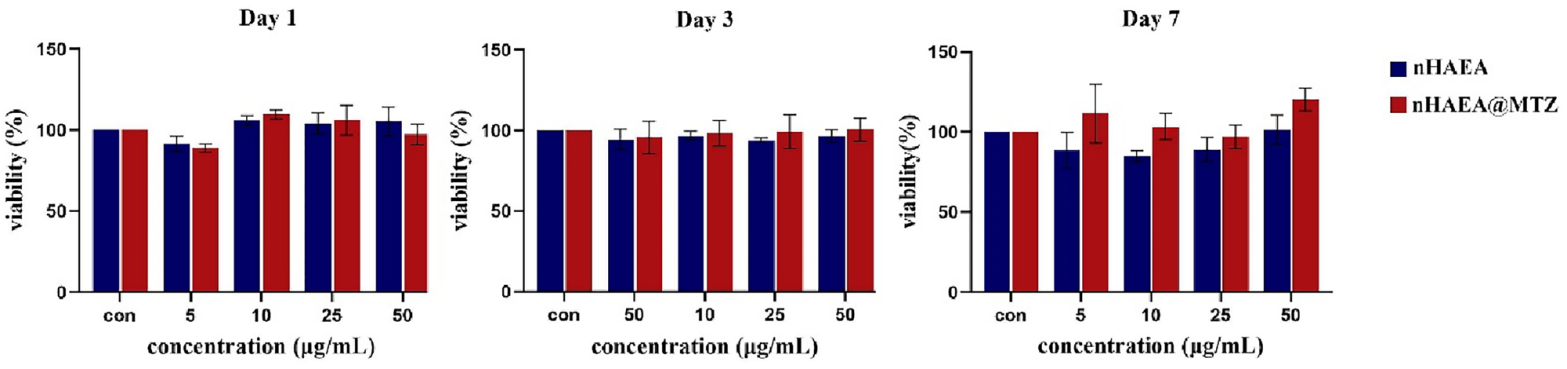
The effect of nHAEA and nHAEA@MTZ on cell viability of dental pulp stem cells after 1, 3 and 7 days. The cell viability was assessed by MTT assay and the percentage of stem cell viability compared to untreated control (100%).

The MTT test was used to assess the viability percentage of HDPSCs treated with LPS and different concentrations of nHAEA and nHAEA@MTZ after 1, 3, and 7 days. This is demonstrated in Fig. 6. MTT colorimetric test assesses mitochondrial activity in cells. At all utilized concentrations and time points, there were no significant differences between the nHAEA-treated or nHAEA@MTZ-treated HDPSCs and the control group. In the following studies, nHAEA and nHAEA@MTZ were used at 50 µg/mL concentration.

### Effects of nanoparticles on cell migration in LPS‐stimulated HDPSCs

**Figure 7:**
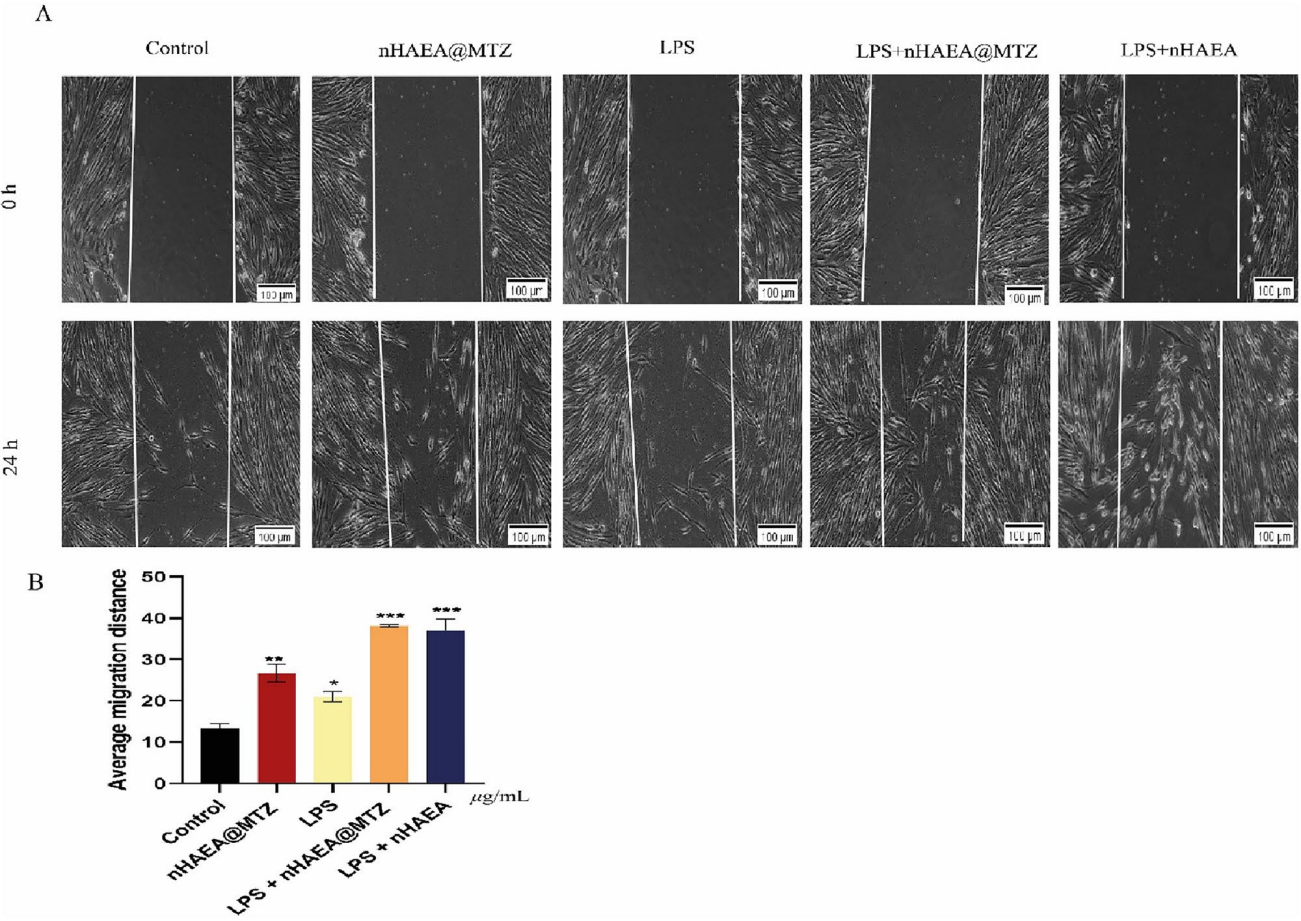
Effects of the nHAEA@MTZ on cell migration under normal and inflammatory conditions. In the scratch test, cells were treated with 50 µg/mL of nHAEA@MTZ with or without 1 µg/mL LPS for 24 h. Micrographs were acquired at 0 and 24 h after scratching. Magnification 10×, scale bar 100 µm. (**A**) The migration of the cells was determined and expressed as the average migration distance after 24 h of treatment (**B**). Each value represents the mean ± SD from three replicate experiments. Statistical analysis was performed using ANOVA ***p < 0.001, **p < 0.01, *p < 0.05. LPS, lipopolysaccharide.

To investigate the impact of NPs on HDPSC migratory capacities, cells were treated with 50 µg/mL nHAEA@MTZ or nHAEA in the presence or absence of 1 µg/mL LPS for 24 h (Fig. 7). The results reveal that treatment with NPs plus LPS increased the migratory ability of HDPSCs compared to control, LPS, or nHAEA@MTZ alone (*p* < 0.001). However, in stimulated HDPSCs is more effective and no significant difference in meaning was observed between the two nanoparticles.

### Anti-inflammatory properties

**Figure 8:**
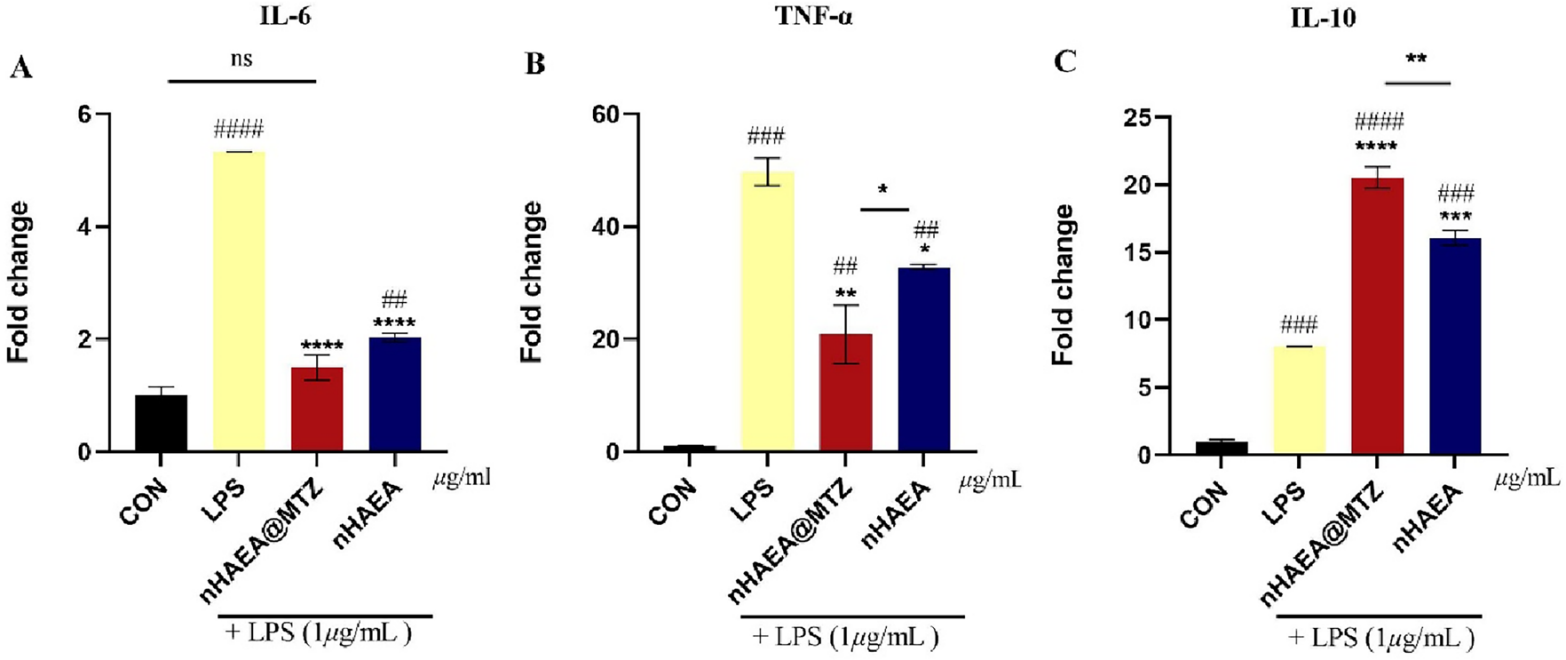
mRNA expression of pro and anti-inflammatory cytokines in HDPSCs stimulated with LPS and treated with nHAEA or nHAEA@MTZ. (**A**) IL-6; (**B**) TNF-α as pro-inflammatory cytokines; (**C**) IL-10 as an anti-inflammatory cytokine. Data are presented as mean ± SD. ^####^*P* < 0/0001, ^###^*P* < 0/001, ^##^*P* < 0.01(vs. untreated control), ****P < 0.0001, ***P < 0.001, **P < 0.01 and *p < 0.05 (vs. group treated with LPS).

In this study, we examined the effects of nHAEA and nHAEA@MTZ on HDPSCs in a simulated inflammatory environment generated by LPS (iHDPSCs). After 24 h of nanoparticle treatment, iHDPSCs' expression levels of IL-10, IL-6, and TNF-α were compared to those of control cells and inflammatory control. We discovered substantial changes in mRNA expression levels of these cytokines between experimental groups (Fig. 8). Stimulating HDPSCs with LPS significantly elevated mRNA expression of IL-6, TNF-α, and IL-10 compared to the control group. Distinctive patterns appeared in groups that received nHAEA or nHAEA@MTZ in addition to LPS simulation. Treatment significantly reduced the expression of pro-inflammatory cytokines IL-6 and TNF-α. On the other hand, the expression of IL-10, an anti-inflammatory cytokine, increased significantly in these treated groups. Notably, the observed changes in cytokine expression were more pronounced in the groups treated with nHAEA@MTZ, suggesting that it has a more regulatory impact on the inflammatory response within HDPSCs under these experimental conditions.

### Odontogenic ability

**Figure 9:**
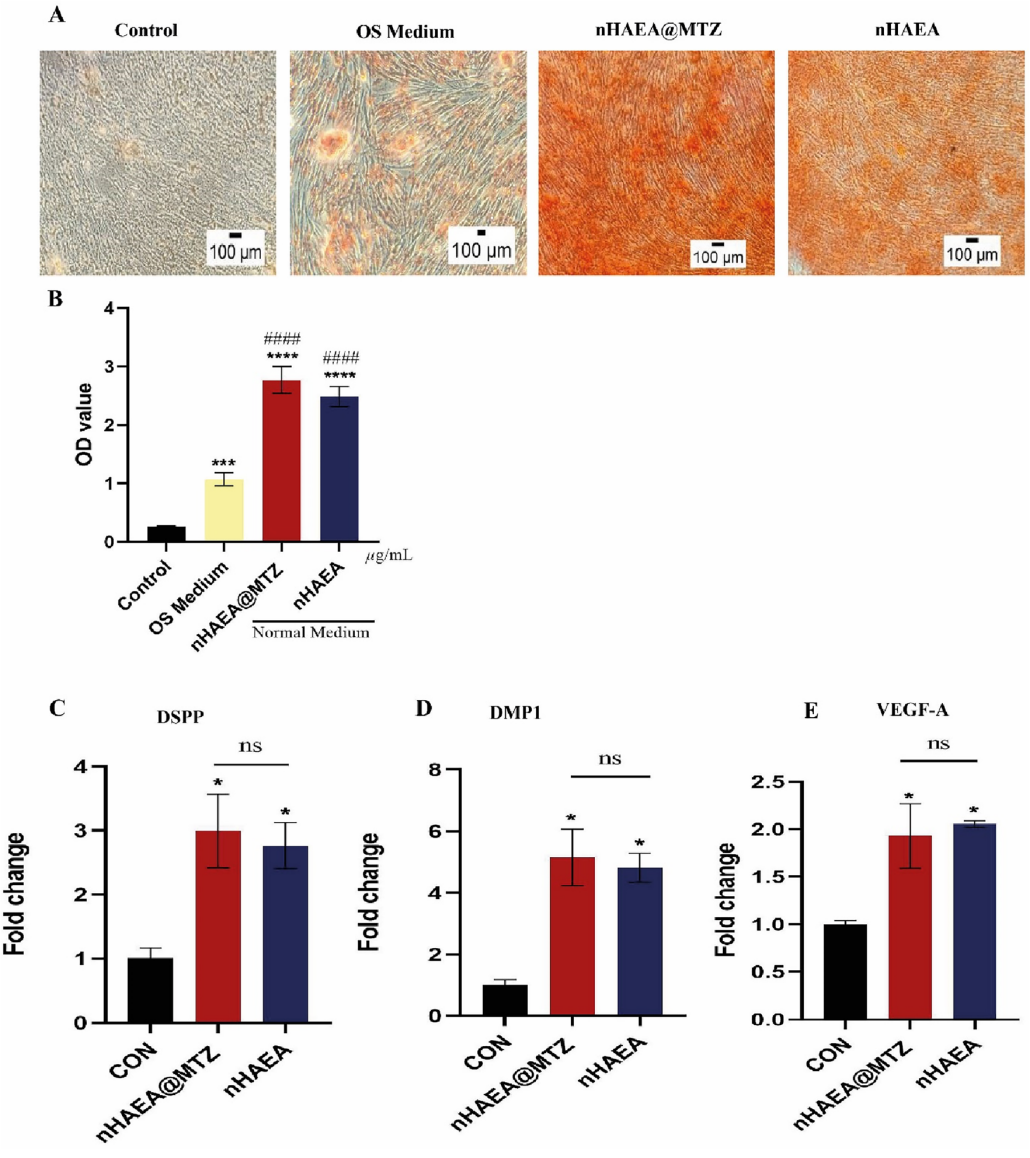
Measurement of osteogenic effects of the nanoparticles on HDPSCs. (**A**) ARS assay of mineralized nodules on day 14 with normal or osteogenic medium. Magnification 10×, scale bar 100 µm. (**B**) Demonstrating quantitative results of differentiated HDPSCs stained with alizarin red S. (**C**) DSPP; (**D**) DMP1; (**E**) VEGF-A. Statistical analysis was performed using ANOVA. ****P < 0.0001, ***P < 0.001 (vs. untreated control with normal medium). ^####^ < 0.0001 (vs. positive control with OS medium), **p < 0.01, *p < 0.05. OS medium; osteogenic medium.

To evaluate the impact of nHAEA@MTZ and nHAEA on the odontoblastic potential of HDPSCs, the cells have been grown in normal medium. For alizarin red staining, cells were treated or not with nHAEA and nHAEA@MTZ for 14 days and also for RT-qPCR analysis cells were treated for 21 days. Alizarin red staining, which indicates the production of mineralized nodules within cell culture plates, revealed that on day 14, the mineralized regions in both treated groups were considerably higher than those in the control group (*P* < 0.05) (Fig. 9A) but no difference was shown between two nanoparticles. Furthermore, quantitative results revealed that cells treated with nHAEA@MTZ exhibited no significantly higher calcium deposition than cells treated with nHAEA (Fig. 9B) (*p* > 0.05). Also, Both NPs markedly increased the mRNA levels of VEGF-A, DSPP, and DMP1 in normal medium without significant differences with each other (*p* > 0.05) (Fig. 9C–E).

## Discussion

Maintaining vital dental pulp following pulp injury is a matter of debate^36^. HDPSCs’ activity is highly connected to the self-healing capabilities of dental tissue injury. Keeping HDPSCs active promotes tooth tissue self-healing following inflammation reduction^37^. Therefore, it is very crucial to reduce inflammation in proper timing and enhance the ability of cells that reside in the tooth pulp to heal itself. Studies showed nHAp has some shortcomings, such as its lowered antibacterial and anti-inflammatory properties^38^. To produce nHAp as a green synthesize to have the ability of drug delivery and anti-inflammatory ability and bone formation, we decided to assess the properties of nHAp synthesized with *Elaeagnus angustifolia L.* extract as drug delivery platforms for metronidazole to treat pulpitis. Two nanoparticles were compared with each other to analyze the effect of MTZ on nHAEA potentials. nHAEA@MTZ conjugates were produced and tested in an in vitro model for the first time. We found that nHAEA can be an efficient drug delivery platform for metronidazole. Furthermore, they have a synergistic anti-inflammatory action and are not harmful to human cells. We used human dental pulp stem cells, which are typically used for studying pulpitis, and assessed their anti-inflammatory properties besides LPS, which is commonly used to induce inflammatory conditions.

Upon examining the FTIR spectrum it was evident that the presence of peaks at 565.86 (υ_4_), 602.8 (υ_4_), and 1034.78 (υ_3_) cm^−1^ signifies molecular vibrations of phosphate $({\text{PO}}_{4}^{3-}$) associated with the structure of hydroxyapatite nanoparticles^39^. Furthermore, the sharp peak at 1034.78 cm^−1^ indicates the stretching mode of the P–O bond in ${\text{PO}}_{4}^{3-}$ (υ_3_)^27,40^. Additionally, the existence of three peaks at 875.52, 1456.99, and 1616.54 cm^−1^ indicates the presence of organic components from *Elaeagnus angustifolia L* extract in the nHAEA structure; where the two peaks at 875.52 and 1616.54 cm^−1^ belongs to the C=O bond, and the peak at 1456.99 cm^−1^ belongs to the C–O bond in the molecular structure of ${\text{CO}}_{3}^{2-}$^40^. Moreover, the expanded peak at 3419.17 cm^−1^ indicates the stretching mode of the O–H functional group, attributed to the absorption of water molecules during the nanoparticle synthesis process^41^. As a result, the production of hydroxyapatite nanoparticles could be confirmed by examining the unique peaks associated with the bonds and functional groups that make up nHAEA. It is also clear that the produced nanoparticles in this study are free of any impurities.

SEM/TEM morphological analysis demonstrated that the nHAEA particles exhibit a nanorod-like structure with a width of 17–23 nm and a length of 93–146 nm. BET is useful in identifying a material's kind of porosity. We demonstrated the curve of nHAEA classified as a type IV isotherm with a hysteresis loop. Mesoporous materials typically display a type IV isotherm, and the graph's shape is related to the capillary condensation process, which usually affects pores with sizes between 2 and 50 nm^42^. Considering the average pore diameter presented in Table 2, the nHAEA particles are of the mesoporous type. Mesoporous particles have a certain size that allows for proper endocytosis and complete transmission as an ideal transporter in regulated drug delivery systems^43^. The mesoporous nHAEA are advantageous for drug loading and release; however, modifying the surface of nanoparticles could greatly increase the loading capacity.

As shown in Table 3, MTZ's loading capacity onto nHAEA nanoparticles was investigated at drug-to-nanoparticle ratios of 1:1 and 2:1. The results showed that at a 2:1 ratio, the percentages of DLC and DLE were higher than the ratio of 1:1. After analyzing various studies, it was clear that the loading amount of MTZ on nHAEA nanoparticles is significantly high. In a study conducted by Sun et al., assessing the loading capacity of ibuprofen on hydroxyapatite nanoparticles modified with triethylamine (HAp + ET), it was determined that the DLE onto HAp + ET nanoparticles was 25.3%^44^. Therefore, the increased loading capacity of MTZ on nHAEA nanoparticles can be due to their highly specific surface area, as evidenced by the BET data. The FTIR spectrum of nHAEA@MTZ clearly shows all peaks attributed to the nHAEA nanoparticle, demonstrating that the hydroxyapatite nanoparticles did not disintegrate after being loaded with the antibiotic metronidazole. Furthermore, the presence of MTZ-related peaks with lower height in the FTIR spectrum of nHAEA@MTZ nanoparticles revealed MTZ embedment within the hydroxyapatite nanoparticles. Therefore, the presence of peaks at 3230.18 cm^−1^ (O–H stretching vibration), 3097.24 cm^−1^ (C–H symmetric stretching), 1537.05 cm^−1^ (N=O asymmetric stretching), 1365.35 cm^−1^ (NO_2_ symmetric stretching), 1263.14 cm^−1^ (C=N stretching vibration), 908.3 cm^−1^ (C–C stretching vibration) and 825.38 cm^−1^ (C–H out-of-plane bending and CN-stretching) can be considered as characteristic peaks of the bonds and functional groups of MTZ, which have appeared in the FTIR spectrum of nHAEA@MTZ^45,46^. It is also anticipated that the potential presence of hydrogen bonds between the OH^-^ groups on the surface of nHAEA and MTZ has led to the entrapment of MTZ antibiotics within the pores of these nanoparticles.

The drug release of the nHAEA@MTZ showed a controlled release profile of MTZ. In normal pH release plateaus were reached after 24 h in which about 40% (w/w) of the loaded drug was released a controlled release profile for the rest of the study period was observed. The quick release is due to MTZ adsorbed on the nanoparticle surface. For acidic pH, an early burst release of 73% was seen, followed by a controlled release for the remainder of the observation period. This was because HAp was degraded to calcium and phosphate ions in acid conditions, which weakened the interaction between HAp nanoparticles and MTZ. Implying that the nHAEA@MTZ had a pH-responsive characteristic for controlling the drug release. Oral Health, which affects oral pH, could impact the potential of nanoparticles^47^. Therefore, it is important to take care of teeth and eat healthy food along with repairing decayed teeth. Liu et al. examined the normal and acidic pH release profile after loading doxorubicin onto hydroxyapatite. At pH 7.4, the nanoparticle release is 39%, while at pH 5.7, it is over 83%. Suggesting that the hydroxyapatite loaded with DOX possessed a pH-responsive property for release^48^. So nHAEA can reduce MTZ’s dosage and reduce its toxic and side effects while maintaining MTZ concentration stably.

Stem cells are essential for regeneration. Therefore, it is critical to increase cell migration capability and hasten the movement of cells to the pulp's deepest location^49^. LPS or nHAEA@MTZ alone increased HDPSC migration, but the combination of the two may accelerate the process even more. In this approach, Chen et al. demonstrate that LPS alone may facilitate cell migration, and a combination of LPS and migratory drugs can increase migration more than either treatment alone^50^.

Adding 1 µg/mL of LPS, a key component of the bacterial outer membrane, to HDPSCs can imitate the in vitro inflammatory environment. This study established an in vitro inflammatory environment model with 1 µg/ml LPS for follow-up investigations^51^. M1/M2 macrophages have been shown to contribute to immunoregulation and reflect the state of inflammation independently by changing polarized activation. Numerous attempts were made to demonstrate that reducing the number of M1 macrophages and enhancing the production of anti-inflammatory cytokines could help to restore homeostasis, which is critical for initiating tissue repair^52,53^. Pro- and anti-inflammatory cytokines, such as IL-10 (a marker of M2 macrophages), TNF-α, and IL-6 (markers of M1 macrophages), increase when LPS mimics an inflammatory environment^49,54^. After treatment, nHAEA and nHAEA@MTZ reduced inflammatory cytokines, indicating that the nanoparticles may have an anti-inflammatory effect. This result was in line with Chen et al.'s use of RvE1 to reduce the expression of TNF-α, IL-1β, and IL-6, three genes linked to inflammation, in LPS-induced HDPSCs. As a result, prompt resolution of pulp inflammation is crucial for protecting the healthy pulp and lowering the rate at which the damaged pulp necrotizes^55^. Also, the anti-inflammatory cytokine IL-10 increased compared to the LPS group, as demonstrated in Li et al. investigation showing THP-1 treatment with FGF-21 with anti-inflammatory properties can increase the amount of IL-10 more than the LPS group^56^. These findings indicate a favorable shift toward an anti-inflammatory response via nanoparticles. In general, NPs changed macrophage polarization from M1 to M2, which is required for pulpitis tissue regeneration. Test results indicate that nHAEA@MTZ has a more pronounced effect, indicating that MTZ works in concert with *Elaeagnus angustifolia L.* Because drugs and nHAEA work together synergistically, the drug may be administered in lower quantities, which may lessen their adverse effects. These findings suggest a promising modulatory impact of nanoparticles on the iHDPSCs, particularly highlighting the enhanced efficacy of nHAEA@MTZ in attenuating pro-inflammatory responses and promoting an anti-inflammatory condition within these cells.

Since dentin makes up most of tooth hard tissue, suitable dentin repair will help protect the pulp and accelerate pulpitis recovery. Dentin is produced and secreted by dentinal cells. As a result, the fundamental components of pulpitis therapy involve promoting HDPSCs to grow into dentin cells and increasing their capacity for mineralization^49^. HDPSCs' osteogenic and odontogenic differentiation is typically assessed by monitoring for the expression of related markers. Several studies examined the expression of osteogenesis-related markers after applying cement or pulp-capping chemicals to dental pulp cells^57^. We investigated the osteogenic effect of MTZ-loaded nanoparticles on HDPSC odontogenic development and compared them to MTZ-free nanoparticles in a non-LPS environment. After 14 days of treatment, the ARS assay revealed increased amount of calcium deposition in the nanoparticle-treated wells in comparison to the control and OS medium (positive control) groups, but no difference was seen between nHAEA and nHAEA@MTZ. In addition, there was no difference in the gene expression of DMP1 and DSPP between the nHAEA and nHAEA@MTZ groups on day 21; these two showed a considerable increase compared to the control group. So, apparently, nHAEA@MTZ has no more influence on osteogenic differentiation. It's worth noting that nanoparticles without osteogenic medium give cells a strong ability to differentiate. In this manner, Chen et al. demonstrated that 4-methylumbelliferone is beneficial for odontoblast differentiation of HDPSCs in normal medium^50^.

The process that mediates the development of the vascular system and angiogenesis is known as vasculogenesis. Tissue regeneration depends on new blood vessels growing from existing vasculature. Numerous research have studied how biomaterials alter vascularization and angiogenesis markers like VEGF in endodontic regeneration^58^. In the current study, we found that nHAEA and nHAEA@MTZ increased VEGF expression on day 21 when compared to the control group. The angiogenesis potential of nHAEA cannot be increased by metronidazole, although both nanoparticles promote VEGF production, which aids in tissue regeneration.

The success of VPT depends on assessing the true pulp status in the clinical setting is challenging and crucial for determining prognosis. The present study is a preliminary investigation into using nHAEA@MTZ as a dental pulp filling material in vital pulp therapy. Further animal studies and additional confirmatory tests are required to explore the role of nHAEA@MTZ in vital pulp therapy more extensively.

## Conclusion

Several physiological activities in HDPSCs, such as cell migration, proliferation, and differentiation, are necessary for tooth repair during inflammation. In the current work, we synthesized nanohydroxyapatite with *Elaeagnus angustifolia L.* extract and, loaded metronidazole on NP for enhancing anti-inflammatory properties (nHAEA@MTZ) and evaluated its efficacy for treating pulpitis. Gene expression showed that nHAEA@MTZ encouraged macrophage M2 polarization by the controlled release of MTZ, and reduced inflammation. This nanoparticle can also enhance the migratory capacity of iHDPSCs. Besides, the nHAEA@MTZ demonstrated acceptable biocompatibility in vitro. Consequently, the green synthesized nHAEA loaded with MTZ can be chosen as an appropriate option for applying pulpitis treatment with additional in vivo research. An in vivo model evaluation of our in vitro results is necessary to comprehend the potential of nHAEA@MTZ properly.

### Supplementary Information

Supplementary Information.

## Data Availability

All data generated or analyzed during this study are included in supplementary information files.
