# Supplementary material for: Green synthesis of nanohydroxyapatite with Elaeagnus angustifolia L. extract as a metronidazole nanocarrier for in vitro pulpitis model treatment
Source: Sci Rep. 2024 Jun 26;14:14702. doi: 10.1038/s41598-024-65582-4 (PMC11208562; doi:10.1038/s41598-024-65582-4)
Supplement: Supplementary file 1 — Supplementary Information. [file 41598_2024_65582_MOESM1_ESM.docx]

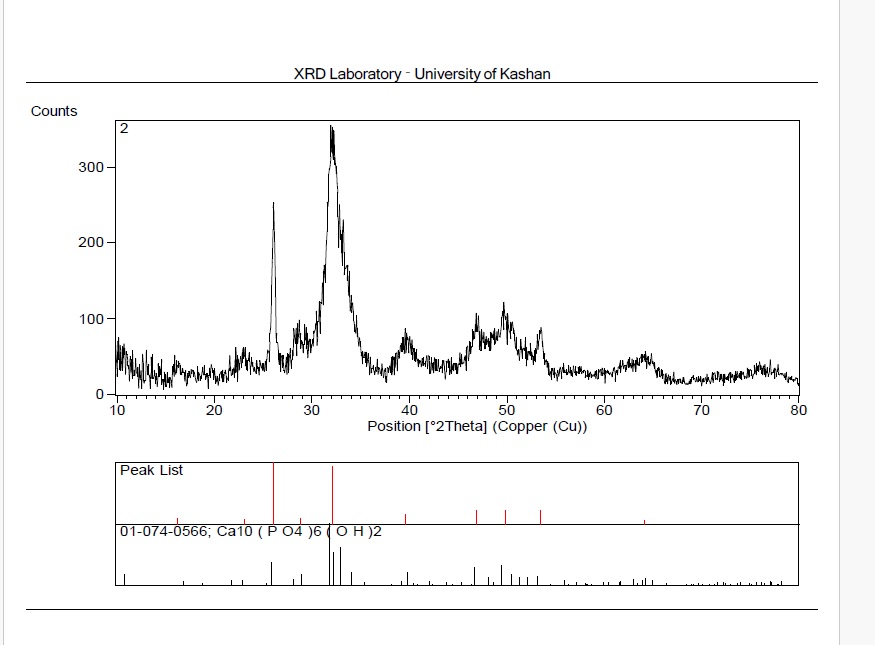


Figure S1: XRD patterns of nHAEA. (Azaryan.2022 doi.org/10.1007/s10266-022-00761-1)

Figure S2: Standard curve of metronidazole in deionized water.

Figure S3: Standard curve of metronidazole in PBS.

Figure S4: Standard curve of metronidazole in acetate buffer.

**Table S1.** Mean and standard deviation (SD) values of cell viability in MTT assay for day 1. nHAEA; nanohydroxyapatite with *Elaeagnus angustifolia L.* extract, nHAEA@MTZ; Nanohydroxyapatite with *Elaeagnus angustifolia L.* extract nHAEA loaded with metronidazole

| SD | Mean | Concentration (µg/ml) | Nanoparticles |
| --- | --- | --- | --- |
| 0 | 100 | Con | nHAEA |
| 4.6 | 91.66 | 5 |  |
| 2.94 | 105.95 | 10 |  |
| 6.3 | 104.2 | 25 |  |
| 9 | 105.5 | 50 |  |
| 0 | 100 | Con | nHAEA@MTZ |
| 2.6 | 89.11 | 5 |  |
| 2.8 | 109 | 10 |  |
| 9 | 106.1 | 25 |  |
| 6.3 | 97.36 | 50 |  |

**Table S2.** Mean and standard deviation (SD) values of cell viability in MTT assay for day 3. nHAEA; nanohydroxyapatite with *Elaeagnus angustifolia L.* extract, nHAEA@MTZ; Nanohydroxyapatite with *Elaeagnus angustifolia L.* extract nHAEA loaded with metronidazole

| SD | Mean | Concentration (µg/ml) | Nanoparticles |
| --- | --- | --- | --- |
| 0 | 100 | Con | nHAEA |
| 6.2 | 94.47 | 5 |  |
| 2.7 | 96.68 | 10 |  |
| 1.19 | 93.97 | 25 |  |
| 3.7 | 96.54 | 50 |  |
| 0 | 100 | Con | nHAEA@MTZ |
| 10 | 95.64 | 5 |  |
| 8.09 | 98.25 | 10 |  |
| 10.4 | 99.38 | 25 |  |
| 7.2 | 100.5 | 50 |  |

**Table S3.** Mean and standard deviation (SD) values of cell viability in MTT assay for day 7. nHAEA; nanohydroxyapatite with *Elaeagnus angustifolia L.* extract, nHAEA@MTZ; Nanohydroxyapatite with *Elaeagnus angustifolia L.* extract nHAEA loaded with metronidazole

| SD | Mean | Concentration (µg/ml) | Nanoparticles |
| --- | --- | --- | --- |
| 0 | 100 | Con | nHAEA |
| 11.1 | 88.55 | 5 |  |
| 3.4 | 85 | 10 |  |
| 7.5 | 89 | 25 |  |
| 8.9 | 101 | 50 |  |
| 0 | 100 | Con | nHAEA@MTZ |
| 18 | 111 | 5 |  |
| 8.1 | 103 | 10 |  |
| 7.2 | 97 | 25 |  |
| 7 | 120 | 50 |  |

**Table S4:** Wound healing sizes in 0 and 24h. Mean and standard deviation (SD) values for the rate of wound closure in this period.

| Hours | Control | | nHAEA@MTZ | | LPS | | LPS + nHAEA@MTZ | |
| --- | --- | --- | --- | --- | --- | --- | --- | --- |
| 0 | 46/31 | 47/52 | 45/25 | 47/12 | 45/58 | 46/97 | 47/47 | 46/23 |
| 24 | 32/11 | 35 | 20 | 18/88 | 23/73 | 26/77 | 9/11 | 8/25 |
| 24-0 | 14/2 | 12/52 | 25/25 | 28/24 | 21/85 | 20/2 | 38/36 | 37/98 |
| Mean | 13.36 | | 26.75 | | 21.03 | | 38.17 | |
| SD | 1.18 | | 2.11 | | 1.16 | | 0.26 | |

**Table S5:** Mean and standard deviation (SD) values of relative gene expression in Real-Time PCR assessment.

| SD | Mean | Groups | Targeted gene |
| --- | --- | --- | --- |
| 0.14 | 1.01 | Con | IL-10 |
| 0.04 | 8 | LPS |  |
| 0.8 | 20.5 | LPS+ nHAEA@MTZ |  |
| 0.55 | 16 | LPS+nHAEA |  |
| 0.14 | 1.005 | Con | IL-6 |
| 0.01 | 5.33 | LPS |  |
| 0.22 | 1.5 | LPS+ nHAEA@MTZ |  |
| 0.07 | 2.02 | LPS+nHAEA |  |
| 0.23 | 1.01 | Con | TNF-α |
| 2.4 | 49.7 | LPS |  |
| 5.1 | 20.8 | LPS+ nHAEA@MTZ |  |
| 0.48 | 32.7 | LPS+nHAEA |  |
| 0.16 | 1.01 | Con | DMP1 |
| 0.9 | 5.1 | nHAEA@MTZ |  |
| 0.47 | 4.81 | nHAEA |  |
| 0.16 | 1.005 | Con | DSPP |
| 0.57 | 2.99 | nHAEA@MTZ |  |
| 0.36 | 2.76 | nHAEA |  |
| 0.04 | 1 | Con | VEGF-A |
| 0.33 | 1.9 | nHAEA@MTZ |  |
| 0.03 | 2.05 | nHAEA |  |

**Table S6:** Mean and standard deviation (SD) values of quantitative results of differentiated HDPSCs stained with alizarin red S.

| Groups | Mean | SD |
| --- | --- | --- |
| Con | 0.26 | 0.01 |
| OS Medium | 1.07 | 0.11 |
| nHAEA@MTZ | 2.76 | 0.22 |
| nHAEA | 2.48 | 0.17 |
